# Does an increased number of cesarean sections result in greater risk for mother and baby in low-risk, late preterm and term deliveries?

**DOI:** 10.12669/pjms.35.1.364

**Published:** 2019

**Authors:** Necati Hancerliogullari, Selen Yaman, Rifat Taner Aksoy, Aytekin Tokmak

**Affiliations:** 1 Necati Hancerliogullari, M.D. Department of Obstetrics and Gynecology, Zekai Tahir Burak Women’s Health Education and Research Hospital, University of Health Sciences, Ankara, Turkey; 2 Selen Yaman, M.D. Department of Obstetrics and Gynecology, Zekai Tahir Burak Women’s Health Education and Research Hospital, University of Health Sciences, Ankara, Turkey; 3 Rifat Taner Aksoy, M.D. Department of Obstetrics and Gynecology, Zekai Tahir Burak Women’s Health Education and Research Hospital, University of Health Sciences, Ankara, Turkey; 4 Aytekin Tokmak, M.D. Department of Obstetrics and Gynecology, Zekai Tahir Burak Women’s Health Education and Research Hospital, University of Health Sciences, Ankara, Turkey

**Keywords:** Cesarean delivery, Intraperitoneal adhesions, Maternal morbidity, Neonatal outcomes, Operative complications, Placenta previa, Repeat cesarean section

## Abstract

**Objective:**

To compare surgical complications and maternal and neonatal outcomes of low-risk, late preterm and term pregnant women who have had one or two previous cesarean sections (CSs) with those who have had three or more CSs.

**Methods:**

We conducted a retrospective study of 850 patients undergoing repeat CS at a tertiary level maternity hospital in Ankara, Turkey. Of those, 380 had previously undergone one or two CSs (Group-I: second or third CS) and 470 had previously undergone three or four CSs (Group-II: fourth or fifth CS). Outcomes and complications were compared between the groups.

**Results:**

The two groups were statistically significantly different in terms of maternal age, parity, body mass index, maternal weight gain during pregnancy, and length of hospital stay (all p<0.001). Although the prevalence of intraperitoneal adhesions and placenta previa was higher in Group-II than in Group-I (p<0.001), there was no statistically significant difference in terms of cesarean hysterectomy and adjacent organ injuries (p>0.05). There were also no significant differences between the groups in terms of neonatal outcomes (p>0.05).

**Conclusion:**

Although the increase in the number of CSs appears to be associated with intraperitoneal adhesions and placenta previa, adverse maternal and neonatal outcomes were not observed in those women with low-risk pregnancies who underwent CS for the fourth or fifth time. Therefore, fourth and fifth CSs may be considered relatively safe surgical procedures in this cohort.

## INTRODUCTION

Cesarean section (CS) is the most common surgery performed in obstetrical practice.^1^ Although it is considered a life-saving procedure in most situations for both mother and infant, CS increases the risk for certain complications, including hemorrhage, injury of adjacent organs, and serious allergic reactions to anesthetic medications. Another significant risk of cesarean delivery is the need for secondary cesarean delivery. Accordingly, repeat cesarean section may increase the risk of morbidity and mortality. A repeat cesarean delivery carries significantly greater risk in terms of abnormal placentation, morbidly adherent placenta, uterine rupture, injury to adjacent organs, excessive blood loss, hysterectomy, and even maternal death.^2^ Many researchers also contend that those risks increase as the number of repeat CSs increases. However, there is no consensus on this issue among published studies to date. Additionally, existing clinical trials are insufficient in determining the maximum number of CSs a woman can safely undergo. Some researchers have found that there is no increase in maternal morbidity in women who undergo three or more CSs and have encouraged these women to consider additional pregnancies.^3^ Select case reports have also documented situations in which women with more than 10 CSs have been safely delivered.^4^

A previous study suggested that surgical delivery may not be a good idea for women planning to have large families.^5^ Subsequent studies found that there is no remarkable difference in serious morbidity associated with multiple repeat CSs.^6^ The safety of anesthesia, the suitable use of antibiotics, and accessibility to safe blood transfusions may render multiple CSs more reliably safe.

In developed countries, where small family size is the standard, women who have had two or three CSs often consider permanent contraceptive options such as tubal ligation. In Turkey, where many refugee women are encouraged through social and cultural influences to have many children, often these families plan for five or six CSs. Another important aspect of this discussion is the neonatal outcomes of these pregnancies. The most commonly discussed issues in studies pertaining to the number of CSs are the causes of maternal morbidity. However, more consistent results regarding neonatal outcomes have been reported.^2^,^6^

Although the safety of CS has been well established, controversy still exists over the number of multiple cesarean sections that is considered safe. Researchers have not clarified whether multiple cesarean sections increase morbidity and mortality. This study sought to compare complications and outcomes of CS in women who had a history of three or more previous CSs with women who had undergone one or two previous lower segment CSs.

## METHODS

We researched the records of women who had previous CSs at Zekai Tahir Burak Women’s Health Education and Research Hospital from January 2016 to December 2016. The hospital’s Institutional Review Board approved the study, but informed consent was not necessary because of the study’s design, which consisted primarily of a retrospective medical record review. Women who met the study’s criteria were identified from a review of the operating room logbook.

In all, 850 women were preliminarily identified, 380 of whom had undergone one or two previous CSs (Group-I), and 470 of whom had undergone three or more previous CSs (Group-II). Patients who did not have a vaginal birth trial and who were contraindicated for labor induction after initial maternal and fetal evaluation were included in the study. Women with a history of classical, T/inverted T shape or low vertical uterine incisions were excluded from the study. Emergency CS was performed on patients who applied during active labor or when fetal distress was detected. All patients were treated with cefazolin IM 1 g before skin incision and were catheterized with a Foley catheter. High-risk pregnancies involving early preterm birth (<35 gestational weeks), chronic hypertension, preeclampsia, intrauterine growth restriction, hyper/hypothyroidism, diabetes mellitus, epilepsy, asthma, chronic inflammatory diseases and multiple pregnancies, were excluded from the study. Pregnant women with isolated placental abnormalities, which are known to be associated with previous CSs, were included in the study.

Data recorded for each patient included maternal age, parity, ethnic origin, body mass index, maternal weight gain during pregnancy, gestational age at birth, length of hospital stay, educational status, preoperative and postoperative 6th hour hemoglobin levels, antenatal, intraoperative and early postoperative complications, and some post-delivery parameters of the neonates. Week of gestation was calculated according to last menstrual period. If patients did not remember the date of their last menstrual period, gestational age was determined through ultrasound screenings carried out in the first trimester. Fetal biometry and Doppler examination by ultrasonography and continuous external fetal monitoring by cardiotocography were carried out for each patient. With 12-hour dosing intervals, 12 mg betamethasone was administered to all pregnant women who were hospitalized at or below 34 weeks of gestation to encourage fetal lung maturation. Placement of infants in the neonatal intensive care unit (NICU) was approved by a neonatologist, and a low Apgar score was defined as below 7 at the 1st and 5th minute following birth. Most patients were seen in regular antenatal follow up, but some were seen in the operating room.

### Statistical Analyses

Statistical analyses were performed using the Statistical Package for the Social Sciences version 22.0 (SPSS, Chicago, IL). Descriptive statistics for normal and non-normal distributed variables were shown as the mean ± standard deviation (SD) and the median (minimum; maximum), respectively. The Shapiro-Wilk test was used to conduct the normality test. To identify differences between the groups, parametric (independent sample t-test) and non-parametric analyses (Mann–Whitney U) were performed following normality analysis. Statistical comparisons between categorical data were carried out by Chi-square test and were expressed as a number (percentage). A P<0.05 was considered statistically significant.

## RESULTS

During the study period, the birth rate was calculated at 18,200 per year in the hospital, and CS was performed in 35% of births. Among the 6,370 CSs performed, 5,520 women were excluded from the final analyses due to primary CS (n:2732), maternal medical conditions (n:1745), early preterm multiple CSs (n: 630), incomplete or untrustworthy data (n:251), unclear gestational age (n:120), and improper incision type (n:42). Mean maternal age was statistically significantly higher in Group-II when compared with Group-I (32.3±4.8 vs. 29.5±5.3 years, p<0.001). There was no difference between the groups in terms of birth weight and gestational week at birth (p>0.05). Additionally, no significant differences were observed between the study groups in terms of educational status, ethnicity, and follow-up programs (p>0.05). Although the rate of placenta previa (both partial and total) was significantly higher in Group-II [16 (4.2%) vs. 53 (11.2%), p<0.001)], there was no significant difference in placenta accreta rates between the two groups (Table-I). The groups were also comparable in terms of patients preoperatively diagnosed with placenta percreta (2 vs. 1). With regard to the newborns in the groups, the rates of both low Apgar scores [23 (6.1%) vs 44 (9.4%), p>0.05] and NICU admission [38 (10%) vs. 40 (8.5%), p>0.05] were similar. 

**Table-I:** Maternal demographics, obstetric and neonatal characteristics.

Characteristics	Group-1 (n:380)	Group-2 (n:470)	P-value
Maternal age in years	29.5±5.3	32.3±4.8	<0.001
Parity	2.4±1.2	3.2±0.6	<0.001
Body mass index	28.6±4.5	29.9±5.0	<0.001
Maternal weight gain during pregnancy in kilograms	13.7±2.8	12.5±2.9	<0.001
Gestational age at delivery in weeks	37.4±1.3	37.3±1.3	NS
Birth weight in grams	3176.8±422.1	3161.3±463.1	NS
Antenatal follow up n (%)	297 (78.2)	357 (76)	NS
Lliterate (n %)	46 (12.1)	65 (13.8)	NS
Refugee (n %)	93 (24.5)	139 (29.6)	NS
Placenta previa (n %)	16 (4.2)	53 (11.2)	<0.001
Placenta acreta (n %)	4 (1)	5 (1)	NS
Placenta percreta n(%)	1(0.3)	2(0.4)	NS
Low apgar score (<7 at 5 minutes) (n %)	23 (6.1)	44 (9.4)	NS
NICU admission (n%)	38(10)	40(8.5)	NS

Data are presented as mean ± Sd, NICU: neonatal intensive care unit, NS: not significant. A p-value<0.05 is considered as statistically significant.

With regard to intraoperative and early postoperative complications of those patients included in the study, intraperitoneal adhesions, the need for balloon application, and duration of hospital stay were significantly higher in Group-II (Table-II). The incidence of tubal ligation was also found to be significantly higher in Group-II when compared to Group-I, as expected. Urgent CS needs of the groups were determined to be similar.

**Table-II:** Intraoperative and early postoperative complications.

Characteristics	Group-1 (n:380)	Group-2 (n:470)	P value
Preoperative hemoglobin mean ± sd	11.6±1.0	11.7±1.1	NS
Postoperative hemoglobin mean ± sd	10.4±1.0	10.5±1.2	NS
Bladder injury	6 (1.6%)	9 (1.9%)	NS
Bowel injury	5 (1.3%)	1 (0.2%)	NS
Caesarean hysterectomy	11 (2.9%)	14 (3.0%)	NS
Blood transfusion	30 (7.9%)	39 (8.3%)	NS
Scar dehiscence or rupture	3 (0.8%)	8 (1.7%)	NS
Emergency Caesarean section	199 (52.4%)	268(57%)	NS
Hospital stay in days	3.5±1.3	4.1±1.5	<0.001
Balloon applications	0	16 (3.4%)	<0.001
Intraperitoneal adhesion	122 (32.1%)	249 (53%)	<0.001
Tubal ligation	173 (45.5%)	288 (61.2%)	<0.001
Intraabdominal drain application	29 (7.6%)	38 (8.1%)	NS
Relaparatomy	2 (0.5)	3 (0.6)	NS

Data are presented as number (percentage), NS: not significant. A p-value<0.05 is considered as statistically significant.

There were no differences between the two groups in terms of preoperative and postoperative hemoglobin levels injuries to neighboring organs and bladder and bowel, blood transfusions, and intraperitoneal drainage procedures (p>0.05). There was also no significant difference between the two groups in terms of uterine dehiscence or rupture from a previous scar site, cesarean hysterectomy, and relaparatomy stemming from advanced complications of cesarean birth (Table-II). There were no instances of maternal mortality cases in either group.

## DISCUSSION

Cesarean section is a surgical procedure frequently used by physicians in obstetrical practice when it is not possible to use normal anatomical routes for delivering babies, or when delivery is urgent. However, like any surgical procedure, it involves various complications and problems, both during and after the operative procedure. Therefore, CS is an important surgical procedure, the complications of which should always be kept in mind. It should only be performed following careful evaluation with carefully executed antenatal follow up, accurate indications for the procedure, and medically appropriate timing. In this study, we sought to compare low-risk pregnant women who have had one or two previous cesarean sections (CSs) with women who have had three or more CSs, specifically examining complications and maternal-neonatal outcomes. According to our study, adverse maternal and neonatal outcomes did not increase in women who have had three or more CSs when compared with women who have had one or two previous CSs.

In examining current surgical practice and literature, we found that the most common complications of repeat CSs are placenta previa, uterine dehiscence or rupture, and vascular and adjacent organ injuries. These complications may lead to high morbidity for the mother during both pregnancy and delivery, and repeat CS procedures raise the level of technical difficulty for surgeons. Of course, it must be noted that among these complications, multiparity alone is a significant risk factor, and this risk is generally considered to increase with repeat CSs. Advanced maternal age was also associated with a range of adverse pregnancy outcomes, regardless of parity.^7^ After controlling for the confounding effects of age and other variables, grandmultiparity has been shown to be associated with some serious obstetric complications.^8^ The majority of studies contend that grandmultiparity can lead to an increased risk of maternal morbidity and mortality due to the increased likelihood of advanced maternal age.^9^

In recent years, CS rates have steadily increased in Turkey and throughout the world. It is interesting to note that Turkey had the highest cesarean delivery rate among the OECD countries, at 53 percent, in 2015.^10^ Nevertheless, medico-legal reservations, the increased safety of CS, and reduced vaginal birth after CS (VBAC) rates play an important role in increasing CS rates.^11^ When compared to first CS and normal birth, repeated CS was shown to be associated with severe maternal and fetal complications.^5^ However, Lynch et al.^3^ reported that repeat CS was associated with low maternal morbidity. They argued the belief that ’“the number of CS a woman undergoes should be limited, and an increased number of CSs raises the risk of complications” is not true. Lynch et al. concluded that pregnancy is safe in women with a history of two or more CSs. Similarly, although dense adhesions and uterine dehiscence are more common, the rates of maternal and fetal morbidity and mortality in women with two or more previous CSs did not significantly differ from those patients with one previous CS.^12^ The rate of three or more cesarean sections in Turkey is not as high.^10^ This may be due to the fact that the culturally desirable number of children may not be high, or health care providers may share with patients their concerns regarding potential problems with recurrent cesarean sections. Undoubtedly, developments in surgical technique, medical devices, anesthetic drugs and intensive care units has resulted in decrease in the incidence of adverse pregnancy outcomes related to CS.

The most dreaded complication of repeat CS is uterine rupture at the previous incisional site. Uterine ruptures can be classified as complete and incomplete ruptures (dehiscence). Full thickness rupture of the uterine wall is defined as complete uterine rupture. Myometrium is usually disrupted in incomplete uterine rupture or dehiscence, but there is no deterioration in the serosa.^13^ Kirkinen^14^ encountered fenestration in 27% of patients who had undergone three or more CS prior to the current operation. However, in subsequent studies, it was reported that those rates ranged between 1% and 10% in patients who had undergone CS five to 10 times. Vaginal birth after cesarean (VBAC) has been initiated as a means of commonly helping to reduce multiple CS rates. The incidence of uterine rupture was found to range between 0.15% and 2.3% during attempted trials of VBAC in women with a low transverse uterine incision.^15^ Scar separation was less visible in our study. Fortunately, we did not observe any symptomatic uterine rupture and maternal mortality. Although elective VBAC is not administered in our hospital, recurrent multiple CS remains a safe procedure without leading to any significant adverse outcomes, as demonstrated in our study.

A study by Al Obaid et al.^4^ compared women with four or more CSs to women with three or fewer CSs in terms of maternal morbidity and complications associated with multiple repeat CS. They found that four or more CSs was associated with a slight increase in maternal complications and surgical difficulties. The duration of surgery and the amount of blood loss were significantly higher, which was possibly the result of the increased prevalence of placenta previa, placenta accreta and peripartum hysterectomy. They concluded that four or more CSs were associated with adverse outcomes, advocating for the importance of careful initial analysis before performing a CS, especially in women who plan to have larger families. Similarly, we found that placenta previa and intrauterine balloon application rates were significantly higher in women who underwent a greater number of CSs.

Choudhary et al.^16^ evaluated operative complications and perinatal outcomes of 224 women who underwent CS two or more times. They found that the incidence of dense adhesions increased with the increasing number of CSs. The incidence of a thin lower uterine segment increased as either the number of CSs or the urgency of the procedure increased. Other intraoperative and postoperative complications did not significantly differ between the groups. The most common adverse fetal outcome they noted was prematurity, which demonstrated an increasing trend in the emergency CS subgroup with two, three, and four or more previous CSs. In our study, no significant difference was observed between the groups regarding uterine dehiscence or rupture, but intraperitoneal adhesions were significantly more frequent in the second group. Additionally, we did not observe significant differences with regard to adverse neonatal outcomes among the groups which was probably due to the exclusion of early preterm pregnancies from the study.

There are signs that postpartum hemorrhage, stillbirth and the risk of uterine rupture increase in subsequent births among patients who underwent planned CSs in their previous births when compared to those who underwent emergency CS.^1^ Gasim et al.^17^ found that blood loss and the need for blood transfusion increased in patients with four or more CSs compared to patients with two to three CS. Yucel et al.^2^ found that multiple CSs (four or more) do not increase the risk of maternal or neonatal complications, with the exceptions of rates of maternal anemia, dense adhesions, and the need for blood transfusion. In contrast to those studies, the need for blood transfusion was not significantly different among the two groups in our study.

In a large retrospective cohort study conducted by Kaplanoglu et al.^11^, patients were separated into five groups, from second CS to sixth CS. As the number of CSs increased, they found that maternal age increased and educational level decreased. Their study also revealed that the duration of the operation and the length of hospital stay were directly proportional to the number of CSs.^11^ Dense adhesions, bladder and bowel injuries, operative complications, and obstetrical complications such as placenta previa, peripartum hysterectomy and blood transfusion have generally been found to be the point of distinction at the fourth CS. There was no difference in terms of postoperative wound infection, endometritis, and wound site dissociation. Placenta adhesion anomalies and the frequency of uterine rupture do not appear to increase with an increasing number of CSs. Uyanikoglu et al.^18^ also compared patients with three or less CSs to those with four or more CS in terms of maternal and perinatal outcomes in their small study population. They did not detect any differences, with the exception of an increase in intraabdominal adhesions. In our study, the prevalence of bladder and bowel injury was found to be low, which is consistent with the extent literature.^2^,^18^ We believe that careful abdominal entry plays an important role in reducing damage to these organs.

A well-established risk factor for placenta previa is previous delivery by CS. The incidence is 2% after one previous caesarean section, 4.1% after two, and 22% after three.^19^ Similarly, surgical interventions to the uterus, such as dilation and curettage, evacuation of the uterus, and myomectomy are associated with placenta previa. It is also more common in older, multiparous women. The reason for this is unclear, but it may be associated with aging of the vasculature system in the uterus. This causes placental hypertrophy and enlargement, which increases the likelihood of the placenta encroaching on a lower segment.^20^ Similarly, the rate of placental invasion abnormalities increases with an increased number of CS. A study by Marshall et al.^21^ found that women with one previous CS had a rate of accreta of 0.3-0.6%. The incidence of accreta continued to rise in accordance with increasing previous cesarean deliveries by as much as 6.74% for women with five or more CSs. Consistent with the extant literature, we found that placenta previa was higher in women undergoing four or more CSs.

### Limitations of the study

It includes its retrospective design, whereas the main strength is that our patient cohort is larger than many studies in the literature. In addition, we believe that exclusion of confounding factors, such as early preterm birth and maternal-fetal medical conditions, known to adversely affect maternal and neonatal outcomes is important in demonstrating the exact effects of recurrent CS on those outcomes. However, the results of the current study were compatible with other studies. We also do not know how the operational characteristics of each patient were recorded. Complications that were considered negligible may not have been recorded. Different teams performed cesarean deliveries, and operative techniques may also differ among practitioners, even if these differences may be minimal.

## CONCLUSION

Our study has demonstrated that the prevalence of intraperitoneal adhesions, placenta previa, and postpartum bleeding problems was higher in low-risk pregnant women with a history of one or two previous CSs than in women with three or more CSs. However, multiple repeat CSs still appear to be a safe procedure for both mother and baby. Patients should be informed about the risks of future pregnancies and possible complications without condemning and discouraging them. There is a need for further prospective studies with a larger sample size.

### Authors’ Contribution

**NH** conceived, designed, did data collection and manuscript writing.

**SY** did statistical analysis and data collection.

**RTA** designed and did data collection.

**AT** conceived, did statistical analysis and editing of manuscript, revised manuscript critically.

All authors approved the final version of the manuscript and agree to be accountable for all aspects of this study.
